# Modulating the Gut Microbiota and Metabolites with Traditional Chinese Medicines: An Emerging Therapy for Type 2 Diabetes Mellitus and Its Complications

**DOI:** 10.3390/molecules29122747

**Published:** 2024-06-09

**Authors:** Peiyan Jiang, Zhenghan Di, Wenting Huang, Lan Xie

**Affiliations:** 1 School of Basic Medical Sciences, Chengdu University of Traditional Chinese Medicine, Chengdu 611137, China; 2 National Engineering Research Center for Beijing Biochip Technology, Beijing 102206, China; 3 Medical Systems Biology Research Center, School of Medicine, Tsinghua University, Beijing 100084, China

**Keywords:** type 2 diabetes mellitus, complications, gut microbiota, metabolites, natural products, traditional Chinese medicines (TCMs)

## Abstract

Currently, an estimated 537 million individuals are affected by type 2 diabetes mellitus (T2DM), the occurrence of which is invariably associated with complications. Glucose-lowering therapy remains the main treatment for alleviating T2DM. However, conventional antidiabetic agents are fraught with numerous adverse effects, notably elevations in blood pressure and lipid levels. Recently, the use of traditional Chinese medicines (TCMs) and their constituents has emerged as a preferred management strategy aimed at curtailing the progression of diabetes and its associated complications with fewer adverse effects. Increasing evidence indicates that gut microbiome disturbances are involved in the development of T2DM and its complications. This regulation depends on various metabolites produced by gut microbes and their interactions with host organs. TCMs’ interventions have demonstrated the ability to modulate the intestinal bacterial microbiota, thereby restoring host homeostasis and ameliorating metabolic disorders. This review delves into the alterations in the gut microbiota and metabolites in T2DM patients and how TCMs treatment regulates the gut microbiota, facilitating the management of T2DM and its complications. Additionally, we also discuss prospective avenues for research on natural products to advance diabetes therapy.

## 1. Introduction

Type 2 diabetes mellitus (T2DM) is one of the most common chronic metabolic diseases and is characterized by an elevated blood glucose concentration and insulin resistance. Abnormal glucose metabolism may lead to multi-organ dysfunctions, and diabetes has become the eighth leading cause of death [1]. According to the International Diabetes Federation (IDF), diabetes was responsible for 4.2 million deaths in 2019. A total of 463 million adults between the ages of 20 and 79 were reported to suffer from diabetes, and this number could increase to 700 million by 2045 [2]. Moreover, in 2021, the global healthcare expenditure for the treatment of T2DM and related complications was US$966 billion, and this number is predicted to exceed US$1054 billion by 2045 [3].

Rapid societal development coupled with unhealthy lifestyles, such as high-fat, high-sugar diets, and excessive alcohol consumption, has increased the prevalence of T2DM [3]. T2DM ultimately leads to diabetic complications, including microvascular (retinopathy, nephropathy, neuropathy) injuries and macrovascular events (such as myocardial infarction, cerebrovascular injuries, and peripheral vascular disease) [4], and cardiovascular disease (CVD) is the leading cause of mortality associated with T2DM [5]. Since T2DM and its complications have a negative impact on the quality of life of patients, shorten their life expectancy, and place a heavy burden on families and society [6], their effective therapeutic management is urgently needed.

Although first-line hypoglycemic drugs are the mainstay of treatment for T2DM, the use of these drugs in the treatment of diabetes is always associated with side effects, including weight gain, high blood pressure, and heart failure [7]. Previous clinical studies have shown that traditional Chinese medicines (TCMs) can alleviate glucose metabolism abnormalities in patients with T2DM without causing serious adverse effects [8,9]. Importantly, treating patients with T2DM with HMs also alleviates diabetes-related complications, such as renal injury [10], osteoporosis [11], and nonalcoholic fatty liver disease [12], and is beneficial for patient quality of life and prognosis. Therefore, in addition to modern therapies, TCMs may be a good choice for treating patients with T2DM.

The gut microbiota is considered the largest microecological system within the host and plays essential physiological roles in host digestion and immunity. Over the past two decades, the gut microbiota has been confirmed to be a key regulator of energy and substrate metabolism in the host. Abnormalities in the composition, diversity, and function of the microbiota result in a disordered metabolic state, including disrupted glucose metabolism and insulin sensitivity [13,14], which are tightly associated with the development and progression of T2DM [15].

Accumulating evidence has indicated that TCMs can closely interact with intestinal bacteria and reshape their composition to improve the microecological environment [16]. By modulating the composition and function of the gut microbiota, TCMs could exert therapeutic effects on T2DM and its complications through mitigating insulin resistance and normalizing disturbed metabolism.

In this review, we first summarize recent research focused on the relationship between the gut microbiota and T2DM and its complications. Additionally, we discuss the therapeutic effects of TCMs on T2DM and its complications through regulation of the gut microbiota based on clinical and animal studies in the last decade.

## 2. The Gut Microbiota Is Associated with T2DM and Its Complications

### 2.1. The Gut Microbiota and T2DM

The gut microbiota (the microbial community in the gastrointestinal tract) has been closely linked with the pathophysiology of most chronic diseases, including T2DM. A disordered gut ecosystem (characterized by changes in the composition of the gut microbiota) increases gut permeability, induces inflammation, and regulates substrate metabolism, thus contributing to insulin resistance [17]. For example, the abundance of *Bacteroides* has been commonly recognized to decrease in T2DM patients [18,19]. Notably, perturbed *Firmicutes*/*Bacteroidetes* (F/B) phylum eubiosis has been frequently observed in T2DM patients [20]. This was also corroborated by animal experiments showing that diabetic mice exhibit a greater abundance of the phylum *Firmicutes* and a lower abundance of *Bacteroidetes* than healthy mice [21]. Similarly, the abundance of *Bifidobacterium* is negatively correlated with T2DM [22]. Detailed studies revealed an increase in the abundance of a specific species, *B. adolescentis*, in patients receiving metformin treatment [23]. The idea that *Bifidobacterium* has a protective effect against T2DM was further supported by animal studies showing that species from this genus (including *B. pseudocatenulatum*, *B. bifidum*, *B. breve*, and *B. animalis*) act as probiotics to improve glucose tolerance in diabetic mouse models [24,25].

*Akkermansia muciniphila*, a mucin-degrading bacterium of the phylum *Verrucomicrobia*, has been reported to be inversely correlated with T2DM [26]. Supplementation of *A. muciniphila* resulted in improved insulin sensitivity and reduced insulinemia [27]. Furthermore, animal studies have demonstrated that an increase in the abundance of *A. muciniphila* could improve insulin resistance and glucose homeostasis while preventing obesity [28,29]. These findings indicate the beneficial effects of *A. muciniphila* on T2DM. For *Lactobacillus*, *L. amylovorus* abundance decreased in T2DM patients, and the abundance of *L. salivarius* increased [30], demonstrating the diversity in the effect of *Lactobacillus* on T2DM. Interestingly, the combination of *Lactobacillus* and *Bifidobacterium* showed a protective effect against T2DM [31].

### 2.2. The Gut Microbiota and T2DM Complications

A series of metabolic disorders associated with T2DM, such as excessive oxidative stress, lipid metabolism, and hypertension, can cause both macrovascular and microvascular complications. CVDs are a kind of macrovascular complication and a leading contributor to the mortality of T2DM patients [32]. Dysbiosis has been shown to facilitate the progression of CVDs. For instance, a high F/B ratio was observed in patients with CVDs. Moreover, relative depletion of the phylum *Bacteroidetes* and the genus *Bacteroides* was reported in patients with heart failure (HF). Another study demonstrated a decreased proportion of *Faecalibacterium prausnitzii* and an increased proportion of the *Enterobacteriaceae* family in patients with CVDs and HF. In terms of the detailed correlation between the gut flora and functional features of CVDs, high abundances of the phylum *Bacteroidetes* and the genus *Bacteroides* were negatively correlated with left ventricular systolic and diastolic dysfunction. Furthermore, left atrial enlargement was shown to be positively associated with the F/B ratio and negatively associated with the abundance of the genus *Bacteroides* [33].

Diabetic nephropathy (DN) is a type of microvascular complication that occurs in nearly 40% of T2DM patients and is the main cause of end-stage renal failure [34]. DN is accompanied by diverse compositional and functional alterations in the gut microbiota [35]. As reported, reduced abundance of *Firmicutes* was observed in mice with diabetic nephropathy [36]. In addition, patients with chronic kidney disease exhibit an increase in the abundance of proinflammatory bacteria, such as *Bacteroidaceae* and *Clostridiaceae*, as well as a decrease in the abundance of anti-inflammatory bacteria (e.g., *Lactobacillaceae*, *Prevotellaceae*, and *Bifidobacteriaceae*) [37].

Diabetic peripheral neuropathy (DPN) is a neurodegenerative complication that has a profound impact on 50% of T2DM patients, and it presents with symptoms of neuropathic pain, numbness, or other paresthesia. Small-fiber neuropathy is the initial pathology of diabetic polyneuropathy. A study revealed that changes in bacterial diversity and a low abundance of *Bacteroides* in the gut were associated with an elevated pain threshold according to intraepidermal electrical stimulation scores [38].

Diabetic cognitive impairment (DCI) is a major complication of T2DM and is caused by constitutive hyperglycemia. Emerging evidence suggests that the gut microbiota and its metabolite SCFAs play important roles in the pathogenesis of DCI. Du et al. found that at the genus level, *Gemmiger*, *Bacteroides*, *Roseburia*, *Prevotella*, and *Bifidobacterium* abundances were higher, and *Escherichia* and *Akkermansia* abundances were lower in DCI patients than in T2DM patients respectively [39].

In summary, the gut microbiota is closely related to the pathology of T2DM and its complications. The mechanisms underlying future treatments for T2DM may involve the regulation of the gut microbiota.

## 3. Metabolites of the Gut Microbiota as Key Factors in T2DM and Its Complications

### 3.1. The Effects of SCFAs on T2DM and Its Complications

In addition to microbial composition, microbial metabolites play key roles in insulin resistance and inflammation and are thus involved in the pathogenesis of T2DM. SCFAs are carboxylic acids produced from the fermentation of indigestible carbohydrates (e.g., dietary fiber) by bacteria in the cecum and colon, and butyrate, acetate, and propionate account for ~95% of the total amount of these SCFAs [40]. Clinical reports have shown that T2DM patients exhibit significantly lower levels of SCFAs in feces than healthy people [41], which is consistent with the results of animal experiments [42].

SCFAs ameliorate T2DM mainly through promoting intestinal gluconeogenesis, reducing fat accumulation, increasing energy expenditure, and reducing inflammation and insulin resistance [43]. For instance, acetate exerts therapeutic effects on T2DM rats through the inhibition of lipogenesis and lipid accumulation in liver and adipose tissue, respectively, as well as the promotion of the transcription of myoglobin and glucose transporter-4 in abdominal muscle [44].

However, a significant decrease in butyrate-producing bacteria and butyrate levels was observed in the guts of patients with T2DM compared to healthy individuals [45], indicating a negative association between butyrate and T2DM. Further studies revealed that butyrate could attenuate inflammation, elevate insulin sensitivity, and enhance mitochondrial function to increase energy expenditure, thereby alleviating T2DM [46,47]. In addition, gut butyrate prevents the proliferation of microorganisms such as *E. coli* and *Salmonella* [48]. Increasing gut butyrate levels, including through the ingestion of dietary fiber, prebiotics/probiotics, and direct supplementation with butyrate preparations, might be an adjunctive therapeutic strategy [46,49].

In addition to their associations with T2DM, SCFAs are closely linked with T2DM complications. SCFAs supplementation can protect against the development of DN [50], as exemplified by the ameliorative effect of sodium butyrate administration on fibrosis in DN animal models [51]. In addition, fecal SCFAs levels were lower in T2DM patients with MCI (mild cognitive impairment) than in normal control individuals and were reported to be negatively associated with Aβ deposition in cognition-related brain regions in the MCI group [52]. The administration of SCFAs could improve cognitive impairment via a microbiota-metabolite–brain axis [53].

### 3.2. Relationships between Branched-Chain Amino Acids (BCAAs) and T2DM and Its Complications

BCAAs (including leucine, valine, and isoleucine) are essential amino acids and exert beneficial nutrient-signaling effects [54]. Paradoxically, BCAAs, the products of microbiota, have emerged as typical biomarkers for a range of metabolic-related diseases, including T2DM [55]. As key factors in glucose and protein metabolism, BCAA levels are elevated in the blood of T2DM patients and are positively associated with insulin resistance [54]. This conclusion is supported by the findings of another study in which individuals with insulin resistance were characterized by an increased abundance of serum BCAAs accompanied by a gut microbiome that has enhanced biosynthetic activity of BCAAs and relative depletion of genes encoding bacterial inward transporters for these amino acids [56]. Although elevated serum levels of BCAAs are associated with insulin resistance, whether this association is causative requires further investigation.

Several studies have shown an association between elevated circulating levels of BCAAs and poor metabolic health. In T2DM patients with stage 1 or 2 chronic kidney disease, high serum BCAA levels are independently correlated with a decrease in the estimated glomerular filtration rate [57]. Another study showed that individuals with both T2DM and Alzheimer’s disease had higher levels of circulating BCAAs and their metabolites than individuals with T2DM alone [58].

### 3.3. Relationships between Bile Acids (BAs) and T2DM and Its Complications

BAs are cholesterol-derived metabolites that function as pivotal signaling molecules that regulate blood glucose and lipid and energy metabolism. Notably, there is a close association between T2DM and BA disorganization. A study revealed higher levels of fasting taurine-conjugated BAs in T2DM patients than in normal glucose tolerance individuals with different levels of insulin resistance [59]. Similarly, in T2DM rats, BAs exhibited characteristic alterations that had negative effects on glucose metabolism [60]. As a bound metabolite of the “host and gut microbiota”, BAs increase glycogen synthesis and maintain blood glucose homeostasis by interacting with Farnesoid X receptor (FXR) and G protein-coupled bile acid receptor 1 (GPBAR-1, also known as TGR5) [61].

BAs also play a crucial role in T2DM complications. BAs are ligands for FXR and TGR5. For DN, it has been reported that the FXR agonist GW4064 can improve functional and structural changes in the kidneys of db/db mice [62]. TGR5 expression and activity is impaired in the kidneys of humans and rodents with obesity and diabetes [63]. TGR5 activation reduces renal inflammatory reactions in diabetic mice, thereby improving renal fibrosis [64].

On another side, the dysfunction of BAs metabolism was found in T2DM-related cognitive dysfunction patients [65]. Song et al. reported that metabolites correlated with disturbances in glucose, lipid, bile acid, and steroid metabolism were significantly altered in db/db mice with cognitive impairment [66]. As another example, mice with diabetes-induced cognitive dysfunction mice have higher BA concentrations in both the liver and ileum than diabetic ones without cognitive dysfunction [67].

The gut microbiota plays a critical role in the metabolism of BAs in the intestinal tract, and alterations in the gut microbiota impact the composition and signaling of the host BAs [68]. The modulation of the structure of the gut microbiota could thus normalize the metabolism of BAs and produce therapeutic effects [43].

This evidence indicates that the intestinal microbiota and its metabolites, including SCFAs, BCAAs, and BAs, are important in the pathology of T2DM and its complications.

## 4. The Gut Microbiota as a Therapeutic Target for Treating T2DM and Its Complications

The involvement of the gut microbiota and its metabolites in the development and progression of T2DM indicates that their modulation is a potential strategy for clinical T2DM treatment. Metformin is an oral blood glucose-lowering compound applied in the treatment of T2DM. It was reported to alleviate hyperglycemic and metabolic dysfunctions by reducing the abundance of *Bacteroides fragilis* and its bile salt hydrolase (BSH) activity, increasing glycoursodeoxycholic acid levels and inhibiting intestinal FXR signalling [69]. Moreover, the increase in beneficial bacteria (such as *Lactobacillus* and *Bifidobacterium*) after metformin administration contributes to its therapeutic effect [70].

Probiotics, generally gram-positive bacteria, are defined as live microorganisms that confer health benefits on human health at an adequate level [71]. The administration of probiotics is another effective approach for regulating the intestinal microbiota in patients with T2DM [72], and the underlying mechanisms involve mitigating hyperglycemia and IR [73]. For example, probiotics isolated from fermented camel milk could enhance glucose metabolism, increase acetic acid levels, and decrease inflammatory cytokines (such as TNF-α and resistin) in T2DM patients [74]. A study with db/db mice demonstrated that these probiotics exerted glycemic control by upregulating GLP-1 secretion and improving the function of the intestinal barrier [75]. In addition, supplementation with probiotic capsules (containing *L. acidophilus*, *L. plantarum*, *L. fermentum*, and *L. gasseri*) for 6 weeks led to a significant alleviation in major diabetic CVD-related parameters in populations with T2DM [76]. *A. muciniphila* is a probiotic that can provide diabetic mice with health benefits by decreasing chronic low-grade inflammation and increasing the production of anti-inflammatory factors (such as α-tocopherol and β-sitosterol) [77].

Prebiotics are consumable substances selectively utilized by microorganisms and confer a benefit to the host [78]. There are several well-known prebiotics, such as inulin, lactulose, fructooligosaccharides (FOS), and galactooligosaccharides (GOS) [79]. Oligofructose-enriched inulin decreased the levels of FPG, glycosylated hemoglobin, and inflammatory markers, including interleukin-6, TNF-α, and plasma lipopolysaccharide, in T2DM patients [80]. Lactulose has been shown to reduce fasting and postprandial glucose and inflammatory marker levels and improve insulin sensitivity [81]. A 3-week lactulose intervention in C57BL/6 mice increased the abundance of the probiotic bacteria *Bifidobacteriaceae* and *Lactobacillaceae*, which increased the α diversity of the gut microbiota [82]. FOS increased GLP-1 levels as well as *Bifidobacteria* and *Lactobacilli* abundances in the caecum of T2DM rats induced by poloxamer-407 (PX-407) [83]. Similarly, supplementation with GOS resulted in an increase in intestinal *Bifidobacterium* abundance and improved fasting blood glucose (FBG) levels in patients with T2DM [84]. However, the effectiveness of prebiotic intervention therapies is also controversial. A clinical study revealed that treating T2DM patients with FOS and GOS for 14 days (16 g/day) decreased the abundance of butyrate-producing bacteria (e.g., *Phascolarctobacterium* in the FOS group and *Ruminococcus* in the GOS group), indicating their adverse effect on T2DM [84].

Fecal material transplantation (FMT), in which stool from a healthy donor is transferred into another patient’s intestinal tract, is an approach for improving microbial diversity and function to correct dysbiosis and has been conducted in clinical and preclinical settings for treatment of T2DM and its complications [85]. FMT can attenuate the apoptosis of pancreatic β-cells, improve insulin resistance, restore intestinal barrier function, reduce plasma glycolipid levels and inhibit chronic inflammation in patients with T2DM and complications [86,87]. For example, Wu et al. revealed that FMT alone and FMT plus metformin can alleviate insulin resistance in patients with T2DM by altering microbial diversity, and the proportions of bacteria negatively correlated with the homeostatic model assessment of insulin resistant states (including *Bifidobacterium adolescentis*, *Chlorobium phaeovibriooides*, and *Synechococcus* sp. *WH8103*) [88].

In general, the gut microbiota and its metabolites are indeed promising targets for the treatment of T2DM and its complications.

## 5. TCMs Intervention in Patients with T2DM and Its Complications Based on the Intestinal Microbiota

### 5.1. TCMs Intervention in Patients with T2DM

#### 5.1.1. Individual TCMs or Chemical Components

**Figure 1:**
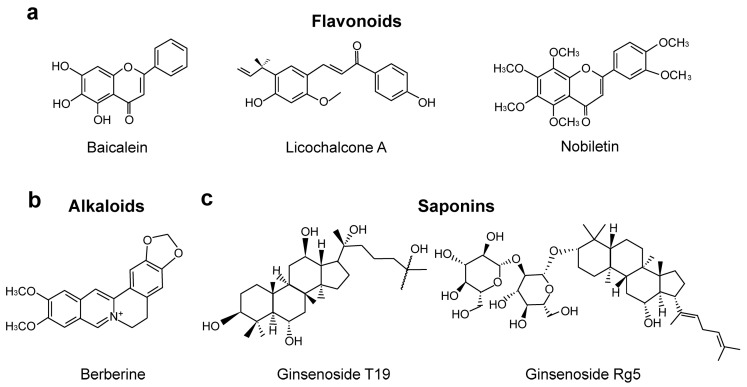
Chemical structures of flavonoids (**a**), alkaloids (**b**), and saponins (**c**) from TCMs that can modulate the intestinal microbiota in T2DM patients.

There is growing evidence that TCMs can alleviate T2DM through regulating the composition of the gut microbiota and reducing inflammation. Herein, we summarized different classes of TCMs, which are grouped according to the bioactive components with which they shift the intestinal microbiota and thus facilitate T2DM alleviation. The antidiabetic mechanisms of these TCMs (Table 1) and the chemical structures of the representative components are further discussed below (Figure 1).

##### Polysaccharides

Polysaccharides are formed by the polymerization of hundreds or thousands of monosaccharide molecules through glycosidic bonds. They are important components of several TCMs and have attracted widespread attention in biomedical research. Notably, polysaccharides have been shown to treat T2DM by modulating the populations and functions of the gut microbiota.

*Ganoderma* (dry fruiting bodies of the fungus *Ganoderma lucidum* or *Ganoderma sinense* (Polyporaceae)), which is named “Lingzhi” in Chinese, is a medicinal mushroom that has been used for centuries to increase vitality and prolong lifespan. Several reports have outlined the benefits of *Ganoderma lucidum* polysaccharides, including immunomodulatory effects, antioxidative effects, anti-inflammatory effects, and antitumor effects [89,90]. Detailed mechanistic investigations using animal models revealed that the administration of *Ganoderma lucidum* polysaccharides to T2DM rats increased the ratio of beneficial bacteria (*Blautia*, *Dehalobacterium*, *ParaBacteroides*, and *Bacteroides*) to harmful bacteria (e.g., *Aerococcus*, *Ruminococcus*, *Corynebactrium*, and *Proteus*) and restored the disrupted carbohydrate, amino acid, nucleic acid, and inflammatory substance metabolism of gut bacterial populations, thus normalizing the gut flora and modifying the metabolism of the host [91]. Furthermore, the hypoglycemic effects of *Ganoderma lucidum* polysaccharides were confirmed by a Phase I/II study in 71 T2DM patients (age > 18 years) who had not received insulin for more than 3 months. The treatment of *Ganoderma lucidum* polysaccharides for 12 weeks significantly decreased the mean HbA_1c_ from 8.4 to 7.6% and the 2 h post-prandial glucose (PPG) levels from 13.6 to 11.8 mmol/L, respectively, while these parameters did not change or slightly increased in patients receiving placebo, demonstrating the hypoglycemic effect of *Ganoderma lucidum* polysaccharides [92].

*Coicis Semen* (Yiyiren in Chinese, dried and mature seeds of *Coix lacryma-jobi* L.var.ma-yuen (Roman.) Stapf (Gramineae)) serves as a hypoglycemic drug, and *Coicis Semen* polysaccharides are the main active ingredient. *Coicis Semen* polysaccharides exhibit glycemic control by promoting the growth of SCFA-producing bacteria, such as *Lactobacillus*, *Akkermansia*, *Bacteroides*, and *Bifidobacterium*, and subsequent activation of the IGF1/PI3K/AKT signaling pathway [93]. Similarly, *Astragali Radix* (Huangqi in Chinese, dry roots of *Astragalus membranaceus* (Fisch.) Bge.var.mongholicus (Bge.) Hsiao or *Astragalus membranaceus* (Fisch.) Bge.(Leguminosae)) polysaccharides increased the populations of SCFA-generating bacteria (*Akkermansia*, *Faecalibaculum*, and *Romboutsia*), elevated the levels of GPCR 41/43, and secreted glucagon-like peptide 1 (GLP-1), leading to a hypoglycemic effect in db/db mice [94].

*Lycii Fructus* (Gouqizi in Chinese, dry and mature fruits of *Lycium barbarum* L. (Solanaceae)), a traditional Chinese medicine (TCM) that has been used for thousands of years, ameliorates hyperglycemia symptoms via *Lycii Fructus* polysaccharides (LFPs). In a high-fat diet (HFD)/STZ-induced T2DM rat model, LFPs alleviated T2DM, which was associated with the reversal of intestinal flora imbalance and abnormal nicotinate/nicotinamide and arachidonic acid/purine metabolism [95]. Further administration of purified homogeneous polysaccharide from crude LFPs ameliorated hyperglycemic symptoms by modulating the composition of the gut microbiota (characterized by a reduced F/B ratio) and the metabolism of SCFAs [96].

*Ophiopogonis Radix*, whose Chinese name is Maidong (Liliaceae), is the tuberous root of *Ophiopogon japonicus* (L.f) Ker-Gawl and has polysaccharides as the main active ingredient. Several kinds of polysaccharides have been extracted from Maidong and shown to have excellent antidiabetic efficacy via intestinal balance and β-cell improvement [97]. For instance, a homogeneous polysaccharide fraction (OJP-W1) significantly improved the glucose tolerance and insulin resistance in diabetic mice by facilitating intestinal microecological balance in HFD-fed mice, as manifested by an increase in *Actinobacteria* and *Bifidobacterium* abundances and a decrease in the abundances of *Proteobacteria* and T2D-enriched taxa (e.g., *Desulfovibrionaceae*, *Dorea*, and *Ruminococcaceae*) [98].

##### Flavonoids

Flavonoids, which contain a basic 2-phenyl-chromone structure, are useful natural compounds that commonly exist in herbs. The pharmacological effects of flavonoids that have gained extensive attention in the industry include antioxidant and free radical scavenging activities [99]. During the past decade, flavonoids have been shown to have therapeutic and preventive effects against T2DM, and these effects are associated with gut microbial metabolism intervention [100].

*Scutellariae Radix* (roots of *Scutellaria baicalensis* Georgi (Labiatae, Huangqin in Chinese)) is a TCM frequently used to alleviate T2DM symptoms (e.g., heat, dampness, and thirst) as well as hyperglycemia. Flavonoids are the chief antidiabetic components of *Scutellariae Radix* and lower blood glucose and lipids by modulating the interaction between the intestinal flora and BA metabolism [101,102]. Notably, baicalein is the most important flavonoid, and it alleviates T2DM by targeting SCFA-producing flora [103]. Baicalein reportedly decreased blood glucose levels and improved insulin resistance, inflammation, and lipid profiles in T2DM rats in a dose-dependent manner. The antidiabetic effects are the result of intestinal microbiota adjustment, followed by an increase in the SCFA concentration and the thickness of the intestinal mucus layer [104]. A clinical study of *Scutellariae Radix* combined with metformin was conducted in 17 eligible subjects (age range of 20–75 years) who had been diagnosed with diabetes ≥ 3 months prior, had an FBG of 110–180 mg/dL or an HbA1c level of 8.0–9.0%, and took ≥ 500 mg/day metformin. The subjects were randomized into *Scutellariae Radix* (*n* = 8) and placebo group (*n* = 9). After 8 weeks of *Scutellariae Radix* or placebo treatment, the indicators of diabetes, including glucose, HbA_1c_, insulin, and homeostatic model assessment–insulin resistance levels, were not changed. However, the blood glucose level after 1 h of an oral glucose tolerance test (OGTT) and OGTT–area under the curve decreased significantly in the *Scutellariae Radix* group compared with the placebo group, indicating that *Scutellariae Radix* plus metformin ameliorated glucose intolerance in subjects. Gut microbiota analysis showed that *Lactobacillus* and *Akkermansia* increased after *Scutellariae Radix* and metformin treatment [105].

Licochalcone A (LicA), a bioactive component of *Glycyrrhizae Radix et Rhizoma* (Gancao in Chinese, roots and rhizomes of *Glycyrrhiza uralensis* Fisch., *Glycyrrhiza inflata* Bat., or *Glycyrrhiza glabra* L. (Leguminosae)), has been found to effectively reverse glucose and lipid metabolism abnormalities and ameliorate T2DM. A mechanistic study revealed that its hypoglycemic activity is the result of the attenuation of intestinal microbiota dysbiosis through the promotion of beneficial bacterial (e.g., *Bifidobacterium*, *Turicibacter*, *Blautia*, and *Faecococcus*) growth and the inhibition of harmful bacterial (e.g., *Enterococcus*, *Dorea*, and *Arachnococcus*) growth [106].

Nobiletin (or polymethoxyflavonoid), which is abundant in orange peels (*Citrus sinensis*, Rutaceae), functions as a hypoglycemic agent through the modulation of the gut microbiota composition, activation of mitophagy flux, downregulation of inflammasome expression, and restoration of islet destruction in the pancreas of an STZ-induced T2DM mouse model [107].

##### Alkaloids

Alkaloids, characterized as nitrogen-containing organic compounds, are indispensable bioactive components of herbs. In T2DM, alkaloids exert hypoglycemic effects chiefly by reorganizing the gut flora structure, promoting glycolysis, stimulating insulin secretion from islet β-cells, and scavenging reactive oxygen species [108].

The excellent therapeutic effect of berberine, a major pharmacological component of *Coptidis Rhizoma* (dry rhizomes of *Coptis chinensis* Franch., *Coptis deltoidea* C. Y. Cheng et Hsiao, or *Coptis teeta* Wall. (Ranunculaceae), Huanglian in Chinese), on T2DM has received increased amounts of attention in clinical research [109,110]. Studies have indicated that berberine exerts glycemic control by modulating the intestinal flora and metabolism of T2DM rats. It increases the populations of *Bacteroidetes*, *Clostridia*, *Lactobacillales*, *Prevotellaceae*, and *Alloprevotella* and reduces the abundance of *Bacteroidales*, *Lachnospiraceae*, *Rikenellaceae*, and *Desulfovibrio* [111]. In addition, berberine could reduce the relative abundance of BCAAs-producing bacteria and serum BCAA levels, therefore effectively reversing glucose intolerance in HFD-fed mice [112].

The novel TCM *Mori Ramulus* (Sangzhi in Chinese, twigs of *Morus alba* L. (Moraceae)) alkaloid tablet (SZ-A) was approved by the China National Medical Products Administration for the treatment of T2DM. A detailed study of diabetic KKAy mice demonstrated that the underlying mechanisms of SZ-A against T2DM lie in the promotion of *Bacteroidaceae* and *Verrucomicrobia*, inhibition of *Rikenellaceae* and *Desulfovibrionaceae*, enhancement of glucose metabolism and the insulin response, and reduction in ileal and systemic inflammation [113]. In a multi-center, randomized, double-blind, double-dummy, and parallel controlled noninferiority clinical trial, 600 patients (age range of 18–70 years) with BMI of 19–30 kg/m^2^, HbA_1c_ level of 7.0–10.0%, and FBG < 13 mmol/L were included in the evaluation of the efficacy and safety of SZ-A, in which they were randomly allocated to the SZ-A group (*n* = 360) and the acarbose (a hypoglycemic drug) group (*n* = 240). After treatment for 24 weeks, the change in HbA_1c_ was −0.93% (95% CI, −1.03 to −0.83) (−10.2 mmol/mol [−11.3 to −9.1]) and −0.87% (−0.99 to −0.76) (−9.5 mmol/mol [−10.8 to −8.3]) in the SZ-A and acarbose groups, respectively, and no significant difference was observed based on covariance analysis between the two groups. Moreover, FBG, 1 h postprandial blood glucose (1 h-PBG), 2 h PBG, and AUC_0–2 h_ in both groups showed a significant decrease from the respective baseline levels. Importantly, the incidence of treatment-related adverse effects and gastrointestinal disorders was significantly lower in the SZ-A group compared to the acarbose group [114]. This study indicated that SZ-A showed equivalent hypoglycemic effects and better safety than acarbose in treating T2DM.

##### Saponins

Saponins are the principal components of many TCMs, including *Ginseng Radix et Rhizoma*, *Platycodonis Radix*, and *Siraitiae Fructus*. The bioavailability of these compounds is insufficient until they are transformed into secondary glycosides and aglycones by the gut microbiota [115]. Both in vivo and in vitro studies have shown that saponins and their secondary metabolites possess antihyperglycemic activity, with effective targets distributed in the intestine [116].

*Ginseng Radix et Rhizoma* (roots and rhizomes of *Panax ginseng* C. A. Mey. (Araliaceae)) is a commonly recognized antidiabetic herb medicine that contains ginsenosides as the main bioactive constituents. Clinical research has confirmed that ginseng treatment significantly decreases FBG levels and improves insulin sensitivity in T2DM patients [117]. For example, ginsenoside T19 administration ameliorates T2DM by reducing the relative abundance of pathogenic bacteria (e.g., *Coprobacillus*) and decreasing the F/B ratio in an STZ/HFD mouse model [118]. Similarly, the ginsenoside Rg5 can reorder the imbalanced intestinal microbiota, mitigate metabolic endotoxemia, inhibit inflammatory pathways (e.g., nuclear factor kappa B (NF-κB) signaling), and restore the intestinal barrier to treat T2DM in db/db mice [119].

*Siraitiae Fructus* (fruits of *Siraitia grosvenorii* (Cucurbitaceae)), termed Luo-Han-Guo (LHG) in Chinese, contains abundant mogrosides and has been used as a folk medicine for the treatment of hyperglycemia in China. In vivo data indicated that 2 weeks of mogroside (extracted from LHG) treatment of STZ/HFD-induced T2DM rats led to the restoration of pathological changes in the gut microbiota, an increase in SCFA concentrations, and a reduction in the content of deoxycholic acid and 1β-hydroxycholic acid in the feces. Importantly, a correlation assay revealed the intestinal microbiota and its metabolites as targets of mogrosides for exerting antidiabetic activity [120].

##### Others

The leaves of *Psidium guajava* (Fanshiliu, Myrtaceae) are a folk medicine frequently used for the treatment of diabetes in Asian countries [121]. To unravel the underlying mechanism, diabetic db/db mice were treated with the aqueous extract of guava leaf (GvAEx) for 12 weeks. GvAEx lowered FBG levels and improved glucose homeostasis and insulin sensitivity, which are associated with alterations in the composition of the microbiota. SCFA-producing *Lachnospiraceae* family and *Akkermansia* genus abundances were increased and the F/B ratio in the intestine was decreased after GvAEx treatment [122].

*Salviae Miltiorrhizae Radix et Rhizoma* (Danshen in Chinese, dry roots and rhizomes of *Salvia miltiorrhiza* Bge. (Labiatae)) is a traditional oriental medicine widely exploited for the treatment of liver and cardiovascular diseases [123,124]. Notably, salvianolic acid A (SalA), a major water-soluble constituent isolated from *Salviae Miltiorrhizae Radix et Rhizoma,* has been revealed to be a potential antihyperglycemic agent. It can attenuate insulin resistance by increasing the abundance and diversity of the gut microbiota and maintaining the balance of the gut core microbiota in Zucker diabetic fatty rats [125].

The extract of *Rhei Radix et Rhizoma* (Dahuang in Chinese, roots and rhizomes of *Rheum palmatum*, L., *Rheum tanguticum* Maxim. ex Balf., or *Rheumoj officinale* Baill. (Polygonaceae)) is commonly used in TCM for treating gastrointestinal diseases. Recent animal experiments have demonstrated its ability to protect mice against high-fat and high-sucrose diet-induced diabetes, and this effect is associated with the promotion of *A. muciniphila* expansion for gut microbiota reorganization [126]. As evidenced by another study, the anthraquinone-glycoside preparation from *Rhei Radix et Rhizoma* enhanced intestinal integrity, thereby reducing the absorption of lipopolysaccharide (LPS) and inflammation in T2DM therapy [127].

Overall, numerous TCMs or bioactive components lower glycemic levels and counteract T2DM progression by modifying the intestinal microecological environment and maintaining the gut flora steady state, which is mostly associated with enrichment of beneficial bacteria and a decrease in harmful bacteria.

**Table 1:** Antidiabetic mechanisms of TCMs and their main bioactive components via gut microbiota modulation. Arrows “↑” and “↓” mean “increase” and “decrease”, respectively.

	TCMs	Bioactive Components	Microbiota Modulation	Mechanisms	Refs.
**Polysaccharides**	*Ganoderma*	*Ganoderma lucidum* polysaccharides	↑*Blautia*, *Dehalobacterium*, *Parabacteroides*, *Bacteroides* ↓*Aerococcus*, *Ruminococcus*, *Corynebactrium*, *Proteus*	Restore the metabolism of gut flora and the host	[91]
	*Coicis Semen*	Coix seed polysaccharides	↑*Lactobacillus*, *Akkermansia*, *Bacteroides*, *Bifidobacterium*	Activate the IGF1/PI3K/AKT pathway, enrich SCFAs-producing bacteria	[93]
	*Astragali Radix*	*Astragali Radix* polysaccharides	↑*Akkermansia*, *Faecalibaculum,* *Romboutsia*	Increase SCFAs, GPCR41/43 and secreted GLP-1 levels	[94]
	*Lycii Fructus*	*Lycii Fructus* polysaccharides	↓F/B ratio	Restore nicotinate/nicotinamide, arachidonic acid/purine and SCFAs metabolism	[95,96]
	*Ophiopogonis Radix*	OJP-W1	↓*Desulfovibrionaceae*, *Dorea*, *Ruminococcaceae*	Facilitate gut microecological balance and β-cell improvement	[97,98]
**Flavonoids**	*Scutellariae Radix*	Baicalein	↑*Bacteroidales S24-7*, *Bacteroidaceae*, *Porphyromonadaceae*, *Verrucomicrobiaceae* ↓*Streptococcaceae*, *Deferribacteraceae*, *Desulfarculaceae*	Adjust intestinal microbiota, increase SCFAs level and intestinal muscus thickness	[103,104]
	*Glycyrrhizae Radix et Rhizoma*	Licochoalcone A	↑*Bifidobacterium*, *Turicibacter*, *Blautia*, *Faecococcus* ↓*Enterococcus*, *Dorea*, *Arachnococcus*	Alleviate gut microbiota dysbiosis	[106]
	Orange peels	Nobiletin	↑*Alloprevotella*, *Parabacteroides*, *Prevotella,* *Desulfovibrio* ↓*Clostridium_XIVa*	Modulate gut microbiota composition, activate mitophagy flux and decrease inflammasome expression	[107]
**Alkaloids**	*Coptidis Rhizoma*	Berberine	↑*Bacteroidetes*, *Clostridia*, *Lactobacillales*, *Prevotellaceae*, *Alloprevotella* ↓*Bacteroidales*, *Lachnospiraceae*, *Rikenellaceae*, *Desulfovibrio*, BCAAs-producing bacteria	Reduce serum BCAAs level	[109,110,111,112]
	*Mori Ramulus*	Sangzhi alkaloid	↑*Bacteroidaceae*, *Verrucomicrobia* ↓*Rikenellaceae*, *Desulfovibrionaceae*	Enhance glucose metabolism and insulin esponse, relieve ileal and systemic inflammation	[113]
**Saponins**	*Ginseng Radix et Rhizoma*	Ginsenoside T19	↑*Coprobacillus*, *Streptococcus*, *Lactobacillus*, *Ruminococcus*, *Anaerotruncus*, *Roseburia*, *Coprococcus* ↓F/B ratio	Ameliorate glucose and insulin tolerance	[118]
		Ginsenoside Rg5	↑*Bacteoidales* ↓F/B ratio	Repress NF-κB signaling, mitigate metabolic endotoxemia	[119]
	*Siraitiae Fructus*	Mogrosides from *Siraitia grosvenorii*	↑*Elusimicrobium*, *Acetitomaculum*	Elevate SCFAs level, reduce deoxycholic acid and 1β-hydroxycholic acid in the faeces	[120]
**Others**	*Psidium Guajava*	Aqueous extract of guava leave	↑*Lachnospiraceae*, *Akkermansia*, *Ruminococcus*, *Anaerotruncus* ↓*Enterorhabdus*, F/B ratio	Reduce gluconeogenesis. enhance glucose uptake and insulin sensitivity	[122]
	*Salvia Miltiorrhiza Radix et Rhizoma*	Salvianolic acid A	↑*Bacteroidetes*, *Proteobacteria* ↓*Firmicutes*, *Tenericutes*, *Verrucomicrobia*	Reduce inflammatory cytokines levels and intestial epithelial barrier injury	[125]
	*Rhei Radix et Rhizoma*	Anthraquinone-glycoside	↓F/B ratio	Rreduce LPS absorption and inflammation	[127]

#### 5.1.2. Chinese Herbal Formulae (CHF)

As the main form of prescription for the clinical application of TCM, CHF have been shown to have therapeutic effects on T2DM via the structural modulation of the gut microbiota. The most well-known antihyperglycemic CHF agents are Gegen Qinlian decoction (GQD), Baihu Jia Renshen decoction (BHRS), Shenling Baizhu powder (SBP), and Shenzhu tiaopi granule (STG).

GQD is a four-herbical agent (*Puerariae Radix* (Gegen in Chinese, roots of *Pueraria lobata* (Willd.) Ohwi, Leguminosae), *Scutellariae Radix*, *Coptidis Rhizoma*, and *Glycyrrhizae Radix et Rhizoma Praeparata cum Melle* (Zhigancao) that has been used for the treatment of diarrhea in Shanghan Lun since the East Han Dynasty. In recent decades, accumulating studies have demonstrated the beneficial effects of GQD in diabetes in both clinical research and animal trials [128,129]. A mechanistic investigation revealed that GQD could enrich populations of butyrate-producing bacteria, including *Faecalibacterium* and *Roseburia*, thus ameliorating inflammation and lowering glucose level [130]. A clinical trial of GQD has been performed in 104 participants, with 50 in the GQD group and 54 in the placebo group, respectively. After treatment for 12 weeks, the HbA_1c_ level in the GQD group was −0.52% [standard deviation (SD): 0.73], which was significantly different (*p* = 0.001) from the placebo group (0.01%, SD: 0.60). Moreover, GQD decreased FPG levels after 4, 8, and 12 weeks with mean changes of 0.61 mmol/L (SD: 1.53), 0.97 mmol/L (SD: 1.44), and 0.94 mmol/L (SD: 1.10), respectively, which were significantly different from the placebo group (with the decrease of 0.10 mmol/L (SD: 1.46), 0.04 mmol/ L (SD: 1.55), and 0.11 mmol/L (SD: 1.45), respectively). Finally, the reduction of the 2 h PBG level in the GQD group (3.42 mmol/L (SD: 3.05)) was significantly higher than the placebo group (0.42 mmol/l (SD 3.76)) at 12 weeks post-treatment. Mechanism investigation demonstrated that GQD increased *Faecalibacterium* (*p* = 0.0153) and decreased *Romboutsia* (*p* = 0.0208) and *Coprococcus* (*p* = 0.0005), while the microbiota composition did not change obviously in the placebo group after 12 weeks. The diabetes-alleviating effect of *Faecalibacterium* was further confirmed by oral administration of *Faecalibacterium prausnitzii* in a T2DM mouse model [131].

BHRS, composed of *Glycyrrhizae Radix et Rhizoma*, *Anemarrhenae Rhizoma* (Zhimu in Chinese, rhizomes of *Anemarrhena asphodeloides* Bge., Liliaceae), Oryzae Sativae Semen (Jingmi in Chinese, seeds of *Oryza sativa* L., Gramineae), *Gypsum Fibrosum*, and *Ginseng Radix et Rhizoma*, was another formula shown to have antidiabetic effects [132]. Inspired by clinical studies showing that BHRS effectively improved insulin sensitivity and lowered blood glucose [133], disease rat models were established via HFD/STZ induction to elucidate the mechanism of BHRS in T2DM. The results suggested that the BHRS-treated group exhibited increased intestinal flora diversity, with increased abundances of *Lactobaculum*, *Blautia*, and *Anaerostipes*, decreased abundances of *Allobaculum*, *Candidatus Saccharimonas*, and *Ruminococcus* and a reduced F/B ratio [134].

SBP (including *Ginseng Radix et Rhizoma*, *Poria* (Fuling in Chinese, dry sclerotia of the fungus *Poria cocos* (Schw.) Wolf, Polyporaceae), *Atractylodis Macrocephalae Rhizoma* (Baizhu in Chinese, roots of *Atractylodes macrocephala* Koidz, Asteraceae), Dioscoreae Rhizoma (Shanyao in Chinese, rhizomes of *Dioscotea opposita* Thunb., Dioscoreaceae), *Lablab Semen Album* (Baibiandou in Chinese, seeds of *lentil Dolichos lablab* L., Leguminosae), *Nelumbinis Semen* (Lianzi in Chinese, seeds of *Nelumbo nucifera*, Nymphaeaceae), *Glycyrrhizae Radix et Rhizoma Praeparata Cum Melle*, *Coicis Semen*, *Platycodonis Radix* (Jiegeng in Chinese, roots of *Platycodon grandiflorum* (Jacq.) A. DC., Campanulaceae), and *Amomi Fructus* (Sharen in Chinese, seeds of *Amomum villosum* Lour., *Amomum villosum* our.var.xanthioides T.L.Wu et Senjen, or *Amomum longiligulare* T.L.Wu, Zingiberaceae), a representative spleen-tonifying prescription, is commonly exploited to treat spleen-deficient diarrhea [135]. In terms of T2DM, SBP increased the relative abundance of SCFA-producing bacteria, including *Bifidobacterium* and *Anaerostipes*, and alleviated chronic inflammation, thereby controlling obesity and alleviating T2DM [136].

STGs, including *Codonopsis Radix* (Dangshen in Chinese, roots of *Codonopsis pilosula* (Franch.) Nannf., *Codonopsis pilosula* Nannf.var.modesta (Nannf.) L.T.Shen, or *Codonopsis tangshen* Oliv., Campanulaceae), *Atractylodis Rhizoma* (Cangzhu in Chinese, rhizomes of *Atractylodes lancea* (Thunb.) DC. Or *Atractylodes chinensis* (DC.) Koidz.), *Dioscoreae Rhizoma*, *Poria*, *Citri Reticulatae Pericarpium* (Chenpi in Chinese, peels of *Citrus reticulata* Blanco, Rutaceae), and *Glycyrrhizae Radix et Rhizoma Praeparata Cum Melle* have been found to prevent insulin resistance and inhibit HbA1c level in T2DM patients [137]. Moreover, STG treatment led to a reduction in FBG levels and a decrease in the F/B ratio. Additionally, the abundances of *Allobaculum* and *Desulfovibrionaceae* were decreased, and the abundance of *Lactobacillus* was significantly increased. These effects are indicative of gut flora modulation for T2DM alleviation [138].

### 5.2. TCMs Ameliorate T2DM Complications by Regulating the Gut Microbiota and Metabolites

#### 5.2.1. TCMs for Treating Diabetic Nephropathy

DN, a frequently occurring microvascular complication, has attracted extensive attention in terms of TCM-based therapy. It has been reported that TCMs treat DN chiefly by modulating the composition of the intestinal flora represented by the F/B ratio, increasing the production of SCFAs and restoring the intestinal barrier. For instance, curcumin, a natural polyphenolic compound derived from turmeric (Jianghuang in Chinese, rhizomes of *Curcuma longa* L., Zingiberaceae), was found to restore the epithelial barrier and reduce LPS-induced renal inflammation by increasing the abundances of gut barrier-friendly bacteria such as *Lactobacillus* and *Bifidobacterium* in the intestines of T2DM patients [139].

Polysaccharides are phytochemicals that act as therapeutic agents against DN through gut microbiota regulation. For example, *Bupleuri Radix* (Chaihu in Chinese, roots of *Bupleurum chinense* DC. or *Bupleurum scorzonerifolium Willd.*, Umbelliferae) polysaccharides improved the dysbiosis of the intestinal flora through decreased blood creatinine and urine albumin levels and inflammatory responses in the kidney and colon in the treatment of STZ-induced DN [140]. *Cordyceps cicadae* (Chanhua in Chinese, Claviciptaceae) polysaccharides ameliorate kidney injury and renal interstitial fibrosis in DN rats by significantly increasing the levels of *Ruminococcus*, *Oscillospira*, and *Roseburia* [141].

In addition to polysaccharides, other bioactive components in TCMs can alleviate DN. As reported, resveratrol could mitigate intestinal permeability and inflammation in db/db DN mice by increasing the abundances of *Bacteroides*, *Alistipes*, *Rikenella*, *Odoribacter*, *Parabacteroides*, and *Alloprevotella*.

A meta-analysis analyzed 25 clinical studies involving 1804 patients with clinical stage III–IV of DN (945 in treatment group and 859 in control group), and the results demonstrated that *Astragalus membranaceus* injection could reduce urea nitrogen, serum creatinine, and urine protein and improve creatinine clearance, thus exerting therapeutic effect on DN [142]. The combination of *Astragalus membranaceus* and *Salvia miltiorrhiza* is an effective prescription for treating DN through increasing the abundances of *A. muciniphila* and *Lactobacillus* murinus and regulating the “gut–kidney axis” [143].

#### 5.2.2. TCMs for Treating Diabetic Cognitive Impairment

Several TCMs are reported to reduce DCI beyond blood glucose control. A polysaccharide extracted from the industrial waste residue of *Astragali Radix* ameliorates ob/ob mouse cognitive impairment by altering the gut microbiota and modulating the composition of metabolites such as SCFAs [144]. Flavonoids from *Astragali Radix* ameliorate brain damage by modulating the brain–gut axis, repairing the blood-brain barrier, protecting hippocampal synaptic function, and improving hippocampal mitochondrial biosynthesis and energy metabolism in HFD/STZ-induced diabetic mice [145].

Ponicidin, a diterpenoid isolated from *Rabdosiae Rubescentis Herba* (Donglingcao in Chinese, aboveground parts of *Rabdosia rubescens* (Hemsl.) Hara, Lamiaceae), decreased the relative abundance of *Firmicutes* and increased the relative abundance of *Bacteroidetes*. Moreover, it restored the relative abundances of the *Allobaculum*, *Lactobacillus*, and *Ruminococcus* genera, which means that it has a neuroprotective effect against diabetic cognitive impairment through modulating the gut microbiome [146].

Compound Danshen dripping pills (containing *Salviae Miltiorrhizae*) restructured the gut microbiota composition and increased intestinal SCFAs in KKAy mice, a model of spontaneous T2DM. These effects may inhibit neuroinflammation, thereby improving cognitive disorders in diabetic mice [147]. A clinical study was performed in 164 DM patients complicated with coronary heart disease, in which they were randomized into two groups, the Fufang Danshen Diwan treatment group and the control group (treated with isosorbide mononitrate). After 16 weeks’ treatment, Fufang Danshen Diwan reduced Aβ levels by 4.31 ± 1.12 pg/mL and improved cognitive function, but no change in Aβ levels was observed in the isosorbide mononitrate group, confirming the effect of Fufang Danshen Diwan to improve diabetic-related cognitive disorders [148].

#### 5.2.3. TCMs for Treating Diabetic Peripheral Neuropathy

Quercetin treatment exerted a protective effect against STZ-induced DPN in rats by modulating the intestinal microbiota and the level of reactive oxygen species, mainly by increasing the level of Actinobacteria [149].

Jinmaitong (JMT), a compound prescription of TCM, has long been used as a therapy for DPN. In STZ-induced DPN rats, JMT may exert neuroprotective effects by modulating the phenotype-related gut microbiota and increasing the serum neuregulin-1 concentration [150]. Clinically, 66 patients with DPN were randomly divided into two groups: the treatment group receiving JMT composita (33 patients with average age of 59.48 ± 8.92 years, FBG value of 9.33 ± 2.77 mmol/L, and 2 h PBG value of 12.15 ± 4.12 mmol/L; 23 cases were complicated with diabetic retinopathy, five with DN; 28 patients were administered with oral hypoglycemic agent, two with insulin, three with traditional Chinese medicine only) and the control group receiving Jingui Shenqi (JGSQ) capsule (33 patients with average age of 58.72 ± 7.77 years, FBG value of 10.57 ± 3.97 mmol/L, and 2 h PBG value of 11.76 ± 4.42 mmol/L; 19 patients were complicated with diabetic retinopathy, four with DN; 27 patients were administered with oral hypoglycemic agent, four with insulin, two with traditional Chinese medicine only). After treatment for 12 weeks, JMT composita significantly decreased red blood cell (RBC) aldose reductase activity, RBC sorbitol, and increased the nerve conductive velocity. However, the amplitude and nerve conductive velocity did not change obviously in the JGSQ group. These results indicated the neuroprotective effects of JMT [151].

Huangqi Guizhi Wuwu Decoction (HGWD) is used to treat blood stagnation and has been used for alleviating DPN in the clinic. This study confirmed that mediating effects on the gut microbiota and plasma metabolism might be the mechanism by which HGWD ameliorates DPN in db/db mice. Moreover, the key underlying mechanism might involve the interactions of *Lactobacillus*, *AlloPrevotella*, *Bacteroides*, and *Desulfovibrio* with sphingolipid metabolism, unsaturated fatty acid biosynthesis, arachidonic acid metabolism, and amino acid biosynthesis pathways [152].

It should be noted that although many TCMs have been confirmed clinically to exert therapeutic effects on T2DM and its complications, the working mechanisms of most TCMs, especially how they exert therapeutic efficacy through gut microbiota modulation, remain unclear and need further exploration.

## 6. Future Perspectives

As shown above, most TCMs can promote the growth of beneficial bacteria and prevent the propagation of harmful bacteria, and the restructured microbiota improves the glucose metabolism of hosts, mainly by exerting anti-inflammatory and antioxidant effects, protecting the intestinal barrier, and inhibiting lipotoxicity. Notably, the bidirectional interaction between TCMs and the gut microbiota is also indispensable for the therapeutic effect of TCMs. That is, TCMs modulate the composition of the microbiota, and alterations in the structure of the microbiota in turn affect the metabolism of TCMs to improve treatment outcomes. In addition, the use of TCMs in combination with Western drugs permits lower doses of the drug and/or decreases the frequency of administration for fewer adverse effects [153].

Since there are similarities in gut microbiome signatures between experimental animals (e.g., mice, rats, and non-human primates (NHPs)) and humans [154], the effects of TCMs on animal models could to some extent predict the therapeutic outcomes in humans. However, the existence of differences in the composition of the gut microbiota between experimental animals and humans (such as a higher *Firmicutes* to *Bacteroidetes* ratio in humans and NHPs vs. mice and rats [154]) make the evaluation of TCMs in the real world necessary. In addition, although TCMs are a treasury of potential prebiotics, we must recognize that the gut microbiota can be impacted by many factors. Among the factors influencing the adult microbiota, dietary factors can account for up to 57% of gut microbiota changes, and recently, the use of a gut microbiota-targeted diet for treating DM has become a topic of widespread concern. Liping Zhao and his colleagues reported that a diet rich in fiber could optimize the gut microbiota, produce more SCFAs, and help to control blood glucose more effectively, suggesting a novel ecological approach for managing T2DM [155]. In addition, the influence of diet on the microbiota may be related to the efficacy of TCMs. Intestinal bacteria can convert herbal chemicals into various bioactive substances, thus promoting better absorption and utilization, while a disordered microbiota may affect the normal metabolism of drugs to some extent. Therefore, we speculate that the gut microbiota shaped by a healthy diet will enhance the efficacy of TCMs, but this hypothesis warrants further investigation.

## Data Availability

Not applicable.
